# Complete Genome Sequence of the Bacillus cereus Temperate Bacteriophage BSG01

**DOI:** 10.1128/mra.00217-23

**Published:** 2023-05-24

**Authors:** Seulgi Imm, Yoonjee Chang

**Affiliations:** a Department of Food and Nutrition, College of Science and Technology, Kookmin University, Seoul, Republic of Korea; Portland State University

## Abstract

The Bacillus cereus phage BSG01 has a siphovirus morphology that can belong to the order *Caudovirales*. It consists of 81,366 bp, with a GC content of 34.6%, and contains 70 predicted open reading frames. BSG01 includes lysogeny-related genes (tyrosine recombinase and antirepressor protein), indicating that it is a temperate phage.

## ANNOUNCEMENT

Bacillus cereus is a Gram-positive, spore-forming pathogen that can cause diarrhea and emetic syndrome (1, 2). Antibiotics have been used to control the pathogen but are generally not recommended because they induce antibiotic resistance problems (3). Therefore, the use of bacteriophages (phages) is emerging as an alternative approach for the control of this pathogen (4). In this study, we report the complete genome sequence of B. cereus phage BSG01, which was isolated from a topsoil sample from Naksan Park (Seoul, South Korea). The phage was isolated by spotting an enriched soil sample on the lawns of a *Bacillus* strain (5). Phage purification and propagation were performed using B. cereus ATCC 27348 as the host bacterium. Briefly, a single plaque was picked and suspended in phosphate-buffered saline (PBS) for phage purification using the phage titration method (6), and this step was repeated three times. Then, the bacterial culture (early exponential phase) was treated with the phage at a multiplicity of infection (MOI) of 1 and incubated at 37°C for 4 h for phage amplification. Phage morphology was confirmed by transmission electron microscopy (TEM) analysis after negative staining with 2% (vol/vol) uranyl acetate (pH 4.5). Furthermore, DNA extraction and purification of phage lysate were performed by phenol-chloroform extraction and ethanol precipitation methods, as described previously (7). The DNA library was constructed using the TruSeq Nano DNA library preparation kit and sequenced with an Illumina MiSeq sequencer (2 × 300-bp paired-end reads). A total of 2,502,910 reads (587,879,675 bp) were trimmed using Trimmomatic v.0.39 (8), and the resulting contigs were *de novo* assembled using SPAdes v.3.13.0 (9) (mean coverage, 855.66×). The open reading frames (ORFs) of the phage genome were predicted using RAST (10), GeneMarkS (11), and FGENESV (5). ORF annotation, phage genome completeness, and phage genome similarity were confirmed using NCBI BLAST (12). The physical ends of the genome were recognized by comparing the sequence identity of the length of the phage.

**FIG 1:**
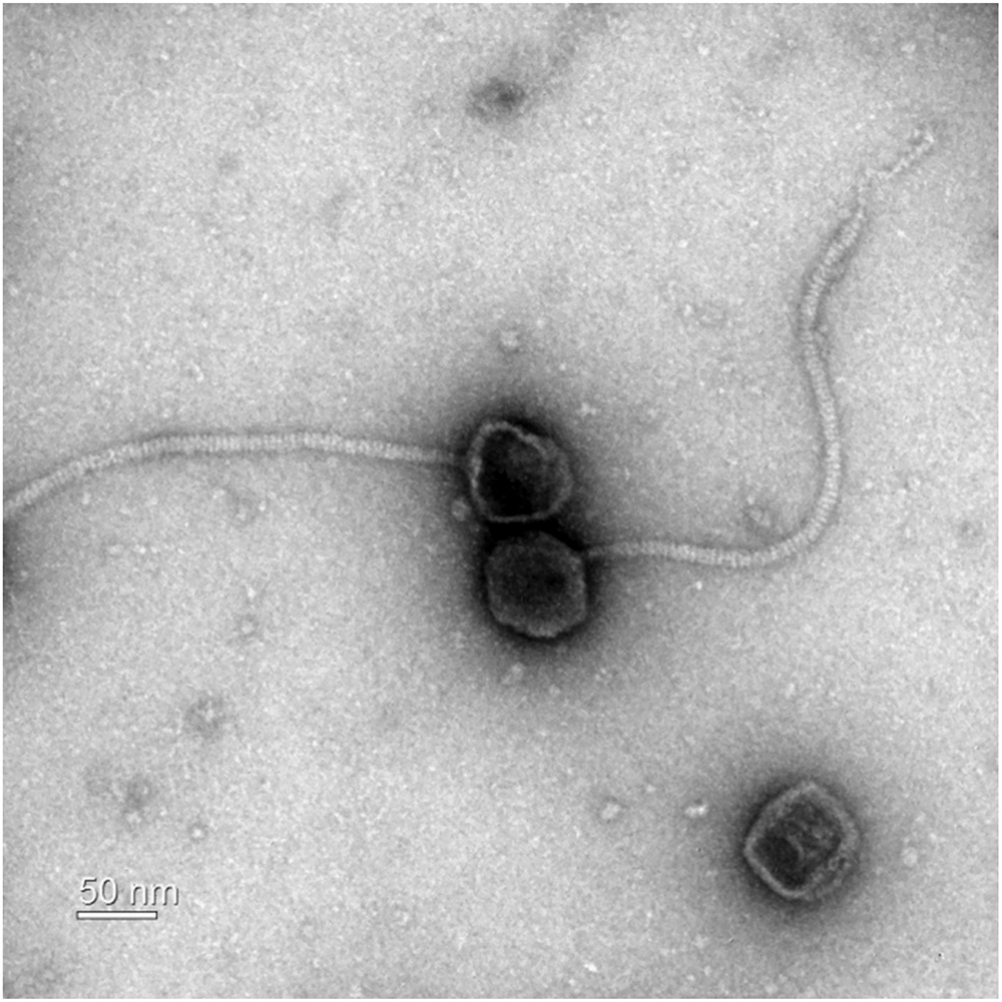
TEM image of phage BSG01 negatively stained with 2% (vol/vol) uranyl acetate. The sample was examined using a Libra 120 transmission electron microscope (Carl Zeiss, Oberkochen, Germany) with an accelerating voltage of 120 kV. Scale bar, 50 nm.

BSG01 has an icosahedral head and a noncontractile flexible tail (Fig. 1). The head diameter was 70.5 ± 0.7 nm (*n* = 5). The tail length and width were 371.0 ± 8.8 nm (*n* = 5) and 10.6 ± 0.2 nm (*n* = 5), respectively. BSG01 has a siphovirus morphology that can belong to the order *Caudovirales* (13).

The complete genome of phage BSG01 consists of 81,366 bp, with a GC content of 34.6%, and contains 70 predicted ORFs. Predicted ORFs were annotated in five groups, including structure and packaging (including terminase-like protein and tail fiber protein), transcription regulation (transcriptional regulator and RNA polymerase sigma factor), DNA replication/modification (including DNA helicase and DNA primase), host lysis (endolysin), and additional functions (including putative peptidase inhibitor and putative GTP-binding protein). Furthermore, the genome was predicted to contain lysogeny-related genes (tyrosine recombinase and antirepressor protein), suggesting that BSG01 is a temperate phage having both lytic and lysogenic life cycles. BSG01 is closely related to *Bacillus* phages PBC4 (GenBank accession number NC_070843), BCP6 (GenBank accession number MW392801), and BCPST (GenBank accession number MW392802), with genome sequence identity of 94% and with percent identity of 96 to 98%.

### Data availability.

The complete genome sequence of phage BSG01 was deposited in GenBank under the accession number OQ436521. The associated BioProject, BioSample, and SRA accession numbers are PRJNA956347, SAMN34211098, and SRR24186942, respectively.
